# Surveillance Routing of COVID-19 Infection Spread Using an Intelligent Infectious Diseases Algorithm

**DOI:** 10.1109/ACCESS.2020.3036347

**Published:** 2020-11-05

**Authors:** Cesar Guevara, Matilde Santos Peñas

**Affiliations:** 1 Centre of Mechatronics and Interactive Systems (MIST)Universidad Tecnológica Indoamérica 185018 Quito 170301 Ecuador; 2 Institute of Knowledge Technology, Complutense University of Madrid 16734 28040 Madrid Spain

**Keywords:** Clustering, computational intelligence, coronavirus disease 2019 (COVID-19), kernel density estimation (KDE), medical care routing, optimization

## Abstract

In this study, the Intelligent Infectious Diseases Algorithm (IIDA) has been developed to locate the sources of infection and survival rate of coronavirus disease 2019 (COVID-19), in order to propose health care routes for population affected by COVID-19. The main goal of this computational algorithm is to reduce the spread of the virus and decrease the number of infected people. To do so, health care routes are generated according to the priority of certain population groups. The algorithm was applied to New York state data. Based on infection rates and reported deaths, hot spots were determined by applying the kernel density estimation (KDE) to the groups that have been previously obtained using a clustering algorithm together with the elbow method. For each cluster, the survival rate —the key information to prioritize medical care— was determined using the proportional hazards model. Finally, ant colony optimization (ACO) and the traveling salesman problem (TSP) optimization algorithms were applied to identify the optimal route to the closest hospital. The results obtained efficiently covered the points with the highest concentration of COVID-19 cases. In this way, its spread can be prevented and health resources optimized.

## I. Introduction

The spread of an infectious disease is a major health problem for citizens worldwide. Identifying the sources of infection is key to stopping a pandemic. It is necessary to act quickly and limit the geographical areas of disease exposure. In November and December 2019, the highly contagious disease known as coronavirus disease 2019 (COVID-19) spread through Wuhan, China. Since then, it has spread to more than 6 million people and 188 countries around the world [1]. Coronaviruses are an extensive family of viruses that can cause disease in both animals and humans. In humans, some coronaviruses are known to cause respiratory infections that can range from the common cold to more serious illnesses, such as Middle East Respiratory Syndrome and Severe Acute Respiratory Syndrome (SARS) [2]. In the case of COVID-19, its rapid expansion has caused many infected people in many countries due to the lack of adequate sanitary resources.

In order to spatially limit the effect of a pandemic, it is necessary to study how infectious diseases spread. The main infection sources (hot spots) and the most vulnerable population areas must be quickly identified. Confinement measures at the national or regional level have proven effective, but these have had strong social and economic impacts in these countries. A more located confinement around infection centers or where there is a higher infection risk could avoid these global measures that negatively affect the economic development of these countries, particularly the more disadvantaged ones.

The objective of this research is to develop a methodology to first locate the regions that are the main sources of infection or may become areas of high infection due to poor sanitary conditions, lack of development, etc. These would allow decision makers to confine only certain sectors of the population that are geographically located at specific areas and thus, to provide the necessary resources so that those areas do not evolve into hot spots due to the lack of health care. Health service routes can be then generated to stop the spread of the virus to other communities. These virus propagation contingency studies are a priority for governments and public health organizations to reduce or prevent the transmission of highly contagious diseases.

In this work, an algorithm to identify the main infection sources (hot spots) from spatial information of reported cases of COVID-19 has been developed. Real data from the United States from February 22 to May 31, 2020 were used. The procedure was as follows. First, the k-means clustering technique was applied using the elbow method to estimate the number of clusters and to group the reported cases spatially. The statistical function known as kernel density estimation (KDE) was used to determine the points with the highest concentration of cases in each of the clusters (hot spots). The algorithm also estimates the survival rate of each of the clusters to determine their care priority. This analysis was performed by applying the proportional hazards model. On the obtained way-points points, the ant colony optimization (ACO) and the traveling salesman problem (TSP) evolutionary algorithms were applied to generated optimal routes to the closest hospital according to the virus survival rates (epidemiological fences).

This combination of intelligent techniques, which are usually applied individually, has been proved to be efficient. For example, in [3] partial derivative regression and nonlinear machine learning are combined for prediction of COVID-19. It obtains an accurate prediction for this pandemic disease in India. The work by Zhang [4] proposes a network-perspective optimization model across multiple social scales (e.g, access, social unbalance, spatial unbalance and resource unbalance) to assign antiviral drugs to the urban dispensing pharmacies in Shangai, China. They apply clustering algorithm, optimization and statistical models.

Our the methodology includes the selection of the most appropriate technique for each of the algorithm’s objectives and the analysis of the configurations that yielded the best results. The generated routes can allow for surveillance and prevention of new virus cases in certain spatial regions. This will facilitate the optimization of both mobility resources (ambulances, cars, etc.) and health facilities (doctors and medicine). In this way, the time until health care arrives is also reduced, which can further decrease the spread of the pandemic. The results obtained in the testing phase were satisfactory; outbreaks of infection were detected with good precision, and the health care routes were optimized.

To summarize, the main contribution of this research is the use of available spatial information on the spread of infectious diseases, such as COVID-19, to improve the care of people affected by the virus and to prevent the spread of any infectious disease. This is way it is possible to develop healthcare routes to optimize hospital resources and prioritize care in the most affected areas. This may have a direct impact on the improvement of sanitary conditions in specific areas as well as in sick patients’ care.

The article is structured as follows. Section 2 summarizes related investigations. Section 3 describes the temporal space information and attributes selection of the U.S. COVID-19 infection dataset. Section 4 presents the proposed Intelligent Infectious Diseases Algorithm (IIDA), and details its four phases. In Section 5, the IIDA algorithm is applied to New York state data, and the results are presented and discussed. The paper ends with conclusions and suggestions for future research.

## II. Related Works

There have been numerous studies on the spread of viruses using simulation models that allow for predicting the evolution of the pandemic over time. However, few works have analyzed spatial expansion. These articles have been limited to indicating the number of infections or deaths by region without providing relevant information that could lead to effective actions, such as local confinement or other measures.

Among the works that have been found in the literature, the one published by Kramer [5] detailed a method for predicting the spread of a disease by evaluating the relative probability of alternative epidemic pathways. This study compared several models that defined the network space movement of the spread of the Ebola virus epidemic in West Africa. The proposed model applied a generalized gravity model using distance and population density to obtain the transmission probability between various cities.

The study conducted by Poon [6] described the implementation of an automated system to monitor and identify hot spots of human immunodeficiency virus (HIV) transmission in British Columbia, Canada. This system used a database that contains more than 32 000 genotypes for almost 9000 residents with HIV. The monitoring system applied clustering of the data to extract groups of five or more individuals with phylogenetic distances.

Gryseels [7] analyzed the spatial spread of yellow fever in Angola and the Democratic Republic of Congo. The author used demographics and human mobility data from Central Africa to predict the spread of the virus. A standard logistic model was used to determine the risk of the virus in each of the districts of the region. The results of the proposed model confirmed that human mobility in high-risk districts significantly influences areas with lower risk.

Wesolowski [8] used mobile phone data to quantify seasonal travel and directional asymmetries in Kenya, Namibia, and Pakistan. The researcher developed a model of the geographic spread of various acute pathogens by applying a time-varying hazard. Furthermore, the model prioritizes the relative importance of routes and their variation throughout the year. In the study by Guzzetta [9], the geographic expansion of dengue in free urban areas was analyzed with spatio-temporal information for Porto Alegre city, Brazil. A Bayesian inference model was applied to geo-located dengue cases from 2013 to 2016. The results showed transmission primarily through short-distance human movement, with some limited contribution from long-distance movements within the city.

Li [10] studied the spatial and temporal characteristics of human H7N9 virus infections in China over a 4-year period. The temporal analysis proved that this virus shows a higher activity at the beginning of the year and then decreases. The space study concluded that the eastern parts of China were more affected initially, and then the virus spread to coastal areas and finally to inland cities in a short period of time.

Migration patterns and their relationship to HIV in 38 communities in Rakai, Uganda were the basis for the study by Grabowski [11]. The researcher used a dataset of 22 000 people with a known HIV status and with a virus prevalence of 9–43%. Migrants moving from a geographical position with a low rate of the disease were found to move to hot spots that had a higher HIV prevalence. However, it was the emigration of people to outside the hot spots that facilitated the geographical spread of the virus. Cumulative distribution functions, medians, and interquartile ranges were applied to obtain the distances traveled by migrant populations. Furthermore, by applying Wilcoxon’s model, the researcher obtained significantly different traveling distances and Shannon entropy based on the geographic diversity between community types.

Nelli [12] analyzed the distribution of malaria in Burkina Faso rural areas, which have a large at-risk population and relatively low accessibility to health services such as hospitals or clinics. This allowed for the prediction of malaria incidence rates based on the distance from health centers. Similarly, Ray [13] used various models to predict the spread of influenza in the United States. In the article published by Nandana [14], the Density-based spatial clustering of applications with noise was applied to disease surveillance. A database of 15 000 cases from Delhi, India in 2011–2013 was used with the aim of reducing the risk of dengue transmission.

Severe acute respiratory syndrome coronavirus 2 (SARS-COV-2) and other coronaviruses have been the focus of more recent investigations. Kang [15] presented the spatial epidemic dynamics of COVID-19 in Mainland China by applying the statistical method known as Moran’s I. This study analyzed spatially close cases to determine if there was a geographical relation between virus infection points. The spatial analysis helped to determine the behavior of infectious disease spread.

The article published by d’Onofrio *et al.* [16] also focused on the spread of an endemic infectious disease. Models were generated that represent changes in people’s social and mobility behavior, such as avoiding visiting areas with a high prevalence of infection. Turing patterns were applied to non-homogeneous SIR models with a prevalence-dependent contact rate. These models responded to spatial variables, mitigation conditions, etc.

Finally, some researchers have studied care routes for populations affected by viruses. For example, Sung and Lee [17] developed a model for the coverage of victims during medical emergencies. This model determines the order in which medical emergencies should be addressed and which destination hospitals these patients should be sent to for care.

In Table 1, the data, models, and methodologies used in different related works are presented. This summary highlights the current state of studies in this area. It can be seen that, in research related to the spread of diseases using spatial data, most of the models are developed by applying time series. The main objective of these works is to monitor the spread of the virus and the location of the infection sources (high number of cases). Few of them make a prediction about the expansion of the virus in the spatial domain. Regarding the techniques used, statistical techniques are applied to the analysis and processing of the data in all of the works. Many of the studies end with this phase of study and analysis, deducing a series of conclusions or suggestions. There are few articles that generate models with these data to make predictions about the spread of infections. Rather, they apply supervised and unsupervised machine learning techniques to this pre-processed data. The main difference between these studies reported in the literature and the one presented here is that the final goal of this study is to determine care routes for infected patients, in such a way as to optimize both care for those infected and health resources. For this reason, evolutionary techniques, specifically Ant Colony Optimization and the Traveling Salesman Problem, are used to determine the optimal healthcare routes, using the survival rate as an optimization criterion.

**TABLE 1:** Comparison of the Methodologies Between Related Works

Research		Data			Model		Methodologies			
Studies	Year	Dataset Name	Alphanum	Spatial	TimeSeries	Predictive	Statistics	Supervised Learning	Unsupervised Learning	Route Optimization
Kramer [5]	2016	Dryad	✔	✔	✔	✔	✔	–	✔	–
Poon [6]	2016	HIV genotype	✔	✔	–	–	✔	–	✔	–
Sung [17]	2016	EMS data	✔	✔	–	–	✔	–	–	–
Gryseels [7]	2017	GenBank	✔	✔	–	–	✔	–	✔	–
Wesolowski [8]	2017	GISDiva	✔	✔	✔	✔	✔	✔	–	–
Guzzetta [9]	2018	Dengue Transmission	✔	✔	✔	–	✔	–	✔	–
Ray [13]	2018	Influenza	✔	–	✔	✔	✔	–	–	–
Li [10]	2019	A(H7N9)	✔	✔	✔	–	✔	–	✔	–
Nandana [14]	2019	Delhi-Dengue	✔	✔	✔	–	✔	–	✔	–
Grabowski [11]	2020	RCCS	✔	✔	–	–	✔	–	–	–
Nelli [12]	2020	Malaria	✔	✔	✔	✔	✔	–	–	–
Kang [15]	2020	Covid-19 China	✔	✔	✔	–	✔	–	–	–
d’Onofrio [16]	2020	–	✔	✔	✔	–	✔	✔	–	–
Guevara	2020	Covid-19 NY, USA	✔	✔	✔	–	✔	–	✔	✔

## III. Dataset Description

Two datasets were used in this study: the COVID-19 database, which determines the rates of infections and deaths worldwide, and a list of Pulmonology and Lung surgery hospitals of the state under study to determine care centers.

### A. COVID-19 Dataset

This data repository collects information on the worldwide spread of Covid-19, which was first identified in Wuhan, the capital of the Hubei province of China. The dataset is compiled by the Center for Systems Science and Engineering at Johns Hopkins University (https://coronavirus.jhu.edu/). This study focused on the country with the highest number of infections (i.e., United States), from February 22 to May 31, 2020. The number of confirmed cases was 1 773 020. The dataset contained 407 625 records and 14 attributes that describe the country coding, geographic location of the infection, and temporal information on positive cases and deaths caused by Covid-19, as shown in Table 2.

**TABLE 2:** Description of Covid-19 Dataset Variables

Variable	Data type	Decimal precision	Type of information	Example
UID	Integer	0	Country coding	84001001
iso2	Text	–		US
iso3	Text	–		USA
code3	Integer	0		840
FIPS	Double	1		60.0
Admin2	Text	–		Barbour
Province_State	Text	–	Geographic location	Alabama
Country_Region	Text	–		US
Lat	Double	6		31.868263
Long	Double	6		−85.387128
Combined_Key	Text	–		Barbour, Alabama, US
Date	Date	–	Temporary information infection and deaths	4/23/20
Confirmed	Integer	0		4
Deaths	Integer	0		1

**FIGURE 1.**
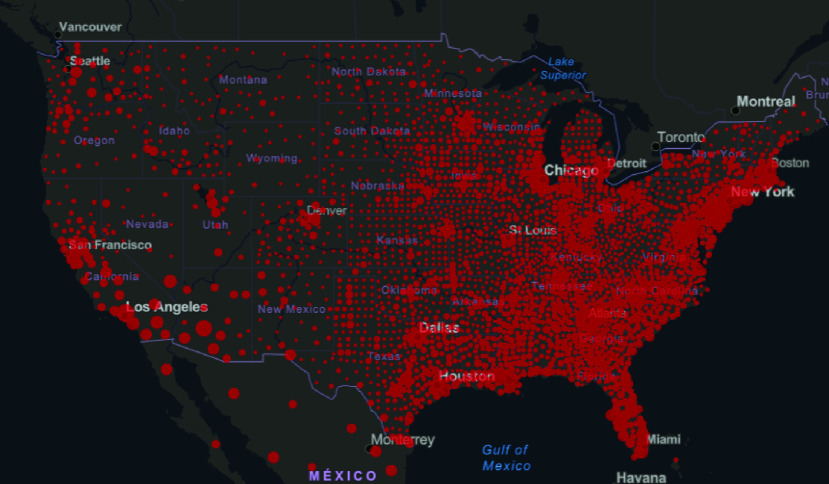
Covid-19 virus infection density in the United States (Johns Hopkins University).

For this study, it was necessary to focus on a specific geographic area, that is, a state with high infection rates within the United States. The five states with the highest confirmed case rates (as of May 31, 2020) were New York with 369 660, New Jersey with 159 608, Illinois with 118 917, California with 109 983, Massachusetts with 96 301, and Pennsylvania with 75 697. The U.S. virus infection density is shown in Fig. 1.

New York was selected because it was the state most affected by the pandemic and has a high rate of mobility of its inhabitants.

### B. Hospital Dataset

A list of hospitals in New York state with Pulmonology and Lung surgery departments was obtained from the New York Department of Health (https://profiles.health.ny.gov). The dataset was comprised of 91 hospitals and their corresponding medical specialty, score, and geographic location (see Table 3).

**TABLE 3:** Description of New York Hospitals Dataset Variables

Variable	Data type	Decimal precision	Type of information	Example
Hospital name	Text	–	Hospital name	North Shore University Hospital
Medical specialty	Text	–	Medical care area	Pulmonology & Lung Surgery
Score	Double	1	Hospital rating for human resources and equipment	77.1
Latitude	Double	6	Geographic location	40.775685
Longitude	Double	6		−73.699704

### C. Features Selection

The most relevant features were selected from the variables presented in Tables 1 and 2. Two techniques, the chi-squared ($X^{2}$) statistical hypothesis test [18] and greedy stepwise algorithm [19], were applied for the attributes selection.

The chi-squared test, defined in (1), was applied to the Covid-19 dataset:

\begin{equation*}X_{c}^{2}=\sum _{i-1}^{k}\frac {(x_{i}-m_{i})^{2}}{m_{i}} \tag{1}\end{equation*}

where $c$ is the degrees of freedom, $x$ represents the observed values for the Covid-19 dataset, and $m$ the expected values. It is supposed that $m$ observations in a random sample from a population are classified into $k$ mutually exclusive classes with respective observed numbers $x_{i}$ (for $i = 1,2,\ldots,k$).

A greedy stepwise algorithm was also applied to the same set to select the most relevant features based on correlation. The result was a percentage of each attribute based on the information provided.

**FIGURE 2.**
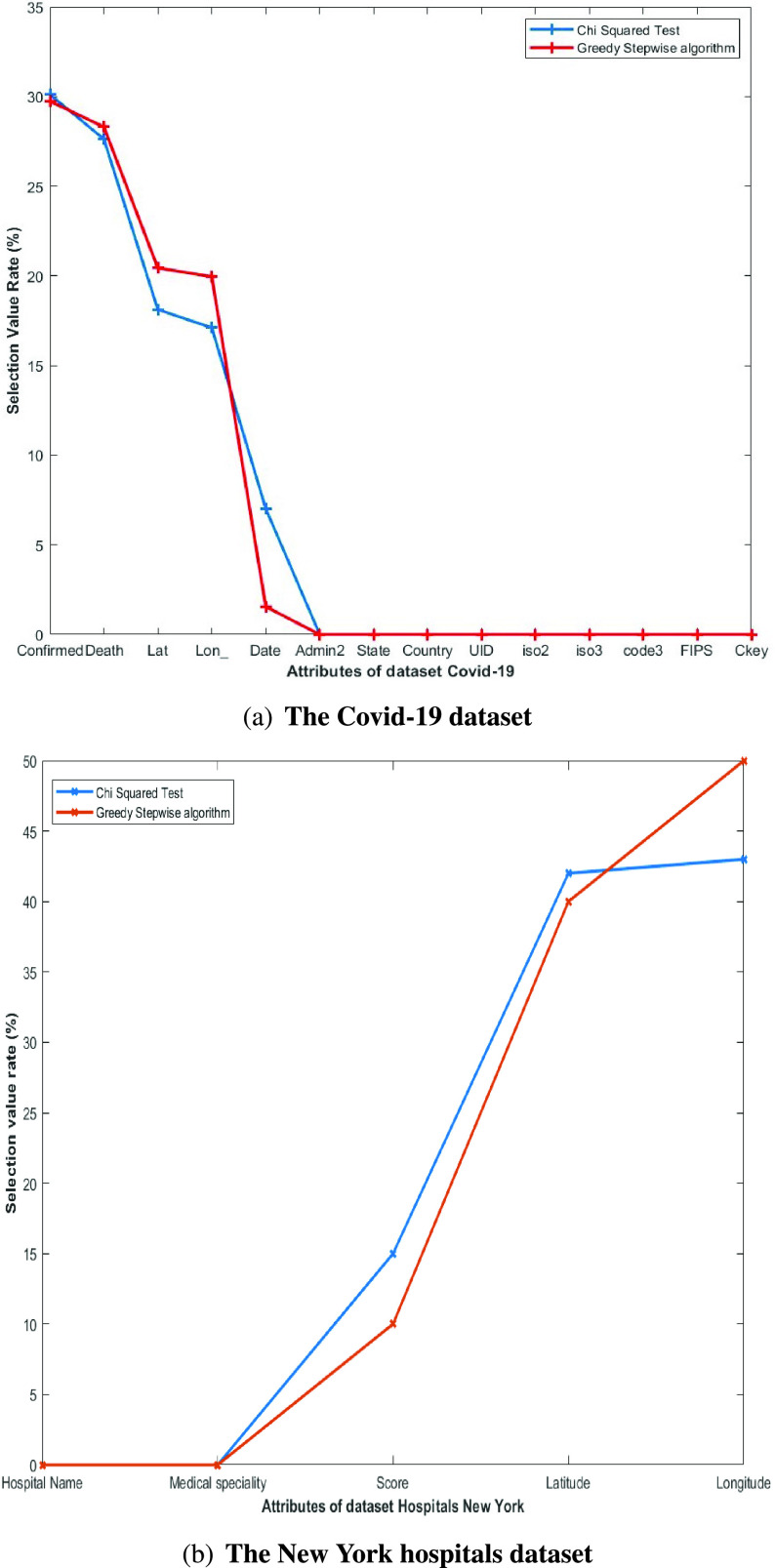
Feature selection with the chi-squared test (blue line) and greedy stepwise algorithm (red line).

The results (in percentages) of the application of the two feature selection methods are shown in Fig. 2 (blue = chi-squared test; red = greedy stepwise). Fig. 2a shows that 5 out of the 14 attributes of the Covid-19 dataset had a value greater than 0. The latitude (Lat) and longitude (Long) values give the geographical location of the cases of infection (Confirmed) and death (Death). The day (Date) provides temporal information for analyzing the spread of the virus. The Death value is used to determine the survival rate in a given area. The Confirmed value determines the infection density. Having selected New York as the state where to apply the IIDA algorithm, the features Admin2, State, and Country do not give any extra information.

For the New York hospitals dataset (Figure 2b), the latitude and longitude were selected since they give the geographic location of the health centers. The attribute score was ruled out because in this state of emergency, all hospitals receive cases of COVID-19 patients.

## IV. Intelligent Infectious Diseases Algorithm

The IIDA was applied to the features selected in previous section. The vector of characteristics is defined as $d_{n} = \{$cf,dt,$t$,lat,long${\$}$. The variable cf represents the number of infected patients, dt is the number of deaths, $t$ is the day they were reported, and lat and long represent the spatial position where infected or deceased patients were identified. The total number of records in New York City for March 2 to May 30, 2020 was 4340. A set of 3064 records from March 2 to May 9, 2020 was selected for training. For testing, a set of 1276 records from May 10 to May 31, 2020 was selected.

**FIGURE 3.**
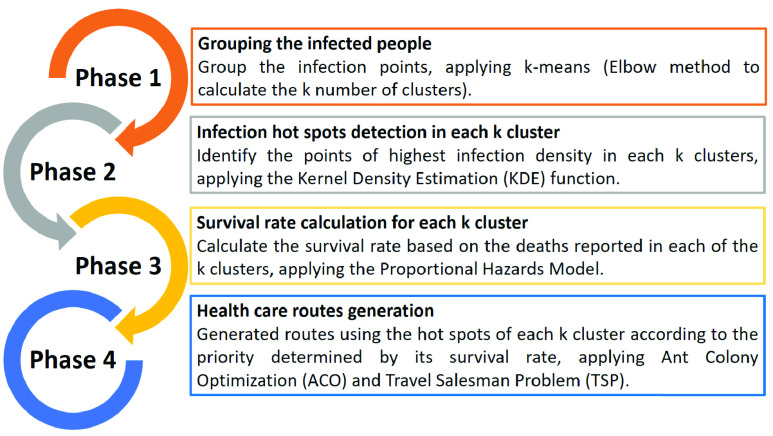
Four stages of the IIDA.

The IIDA algorithm was applied in four phases, which are described in Fig. 3. Details for each phase are explained below.

### A. Phase 1: Spatial Grouping of the Infected Cases

In this phase, the main objective was to spatially group cases of COVID-19 infection in New York state. The k-means clustering algorithm, one of the most commonly used algorithms among partitional methods, was applied [20].

The standard k-means algorithm follows an iterative methodology. First, $k$ points are randomly selected and used as initial means of $k$ clusters. Then, each point in the dataset is assigned to the cluster with the nearest mean. The goal is to minimize the within-cluster sum of squares (SS_*w*_) calculated by

\begin{equation*} {{\mathrm {SS}}}_{w}=\sum _{i-1}^{k}{\sum _{d_{n}{(t)_{j}\in S_{i}}}{||d_{n} (t)_{j}-\mu _{i} ||^{2}}} \tag{2}\end{equation*}

where $x$ represents observations, $S$ represents clusters, and $\mu _{i}$ is the mean of observations in cluster $S_{i}$. The sum of squares is the squared Euclidean distance; therefore, choosing the nearest mean will generate the minimum SS_*w*_. Once all data points are assigned to the $k$ clusters, the SS_*w*_ is calculated, and the new centroids of the clusters are identified and used as new means. The point assignment and mean update steps repeat until the minimum SS_*w*_ is reached.

**FIGURE 4.**
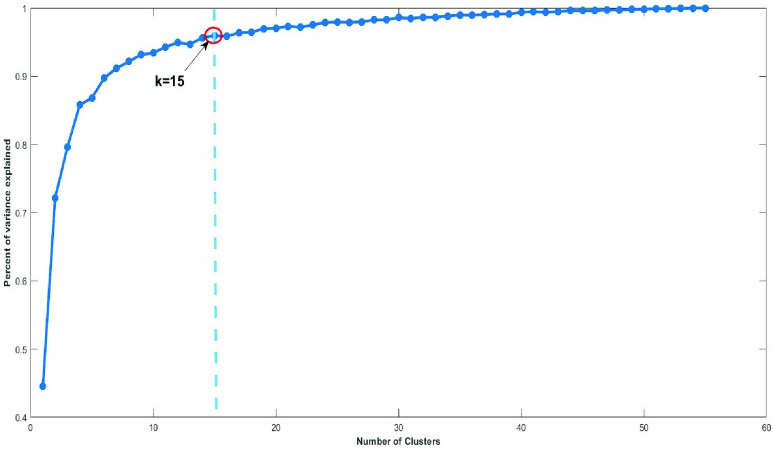
Application of the elbow method to obtain the optimum number of clusters ($k=15$).

The number of clusters $k$ should be predefined for the k-means algorithm; however, there are different clustering evaluation criteria that can be used to estimate the optimum number of clusters. In this phase, different combinations of common cluster analysis criteria were used to determine if a fully-automated clustering of the COVID-19 dataset was feasible. Finally, the elbow method [21] was used to determine the best partition. The elbow method computes the clustering algorithm for different values of $k$. Then, for each $k$, it calculates the total SS_*w*_. The representation of SS_*w*_ regarding the number of clusters $k$ allowed us to find the correct number of clusters (Fig. 4).

**FIGURE 5.**
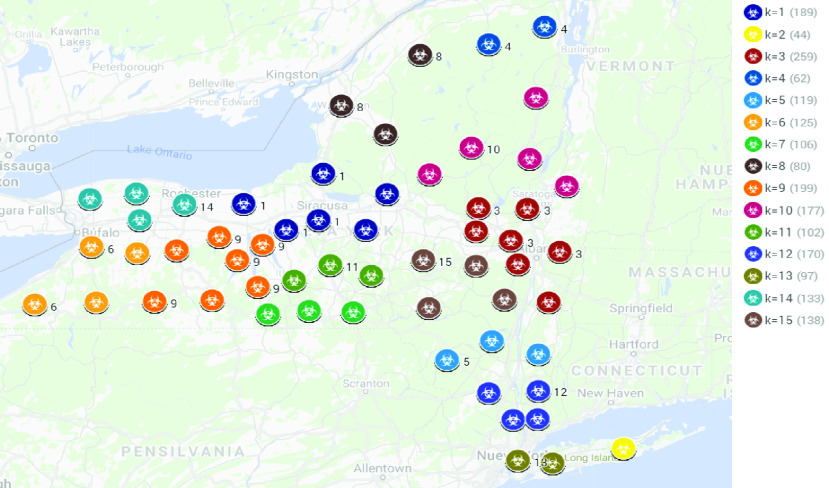
K-means clustering ($k = 15$) in New York state.

Once the optimum number of clusters is obtained, in this case $k = 15$, the k-means algorithm is applied. Fig. 5 shows the results for New York state. Each colored circle represents a different number of points (between bracket). The color indicates the cluster (from $k = 1$ to $k = 15$).

After the optimal number of clusters has been identified, it is added as an attribute, $c_{k}$, to the characteristics vector, i.e., $d_{n} = \{$cf,dt,$t$,lat,long,$c_{k}\}$.

### B. Phase 2: Infection Hot Spots Detection in Each $k$ Cluster

The objective of this phase was to identify the hot spots in each of the clusters generated in Phase 1. The KDE function is a non-parametric method to estimate the probability density function of a random variable [22]. A popular version of this type of methodology is the sample point adaptive density estimator, defined by

\begin{equation*} \hat {f}_{h}(x)=\frac {1}{n} \sum _{i=1}^{n} K_{h} (x-x_{i})=\frac {1}{nh} \sum _{i=1}^{n} K \frac {x-x_{i}}{h} \tag{3}\end{equation*}

where $x_{1},\ldots,x_{n}$ are the bivariate coordinates of $n$ independent, identically distributed observations; $K$ is the kernel (a non-negative function); and $h$, a smoothing parameter called the bandwidth, is greater than 0. A kernel with subscript $h$ is called the scaled kernel and defined as $K_{h} (x)=\frac {1}{h} K\left({\frac {x}{h}}\right)$.

**FIGURE 6.**
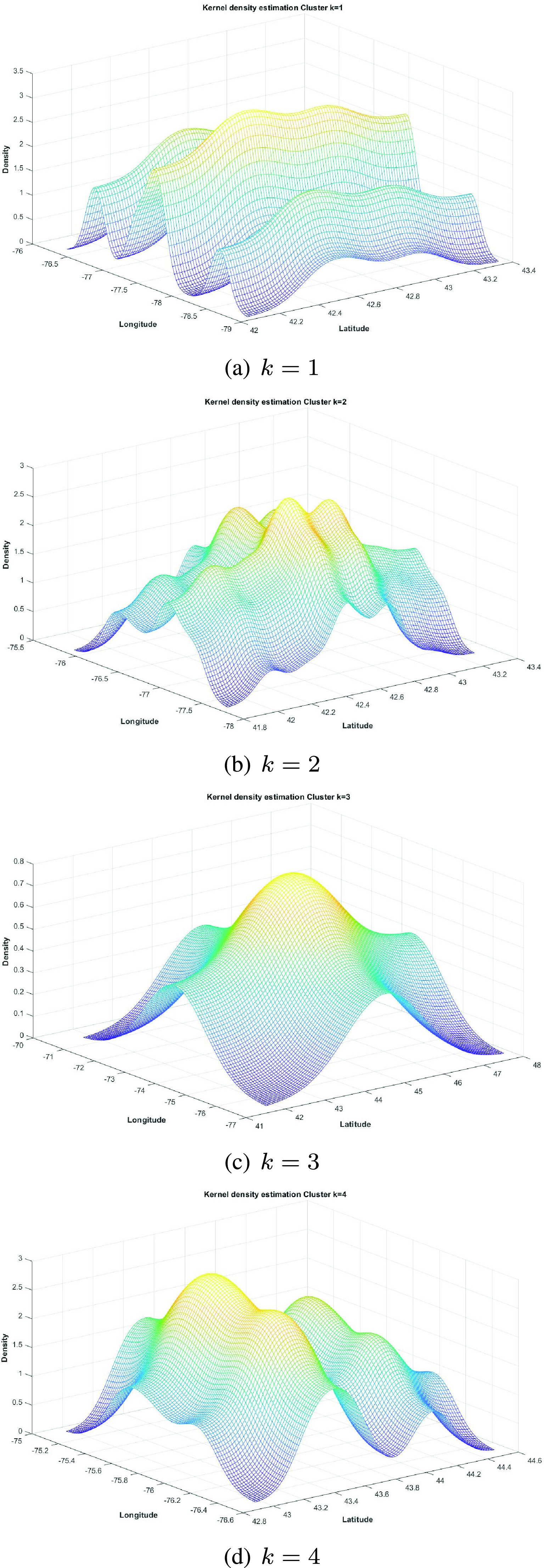
Hot spots obtained with KDE for clusters $k = 1$ to $k = 4$.

**FIGURE 7.**
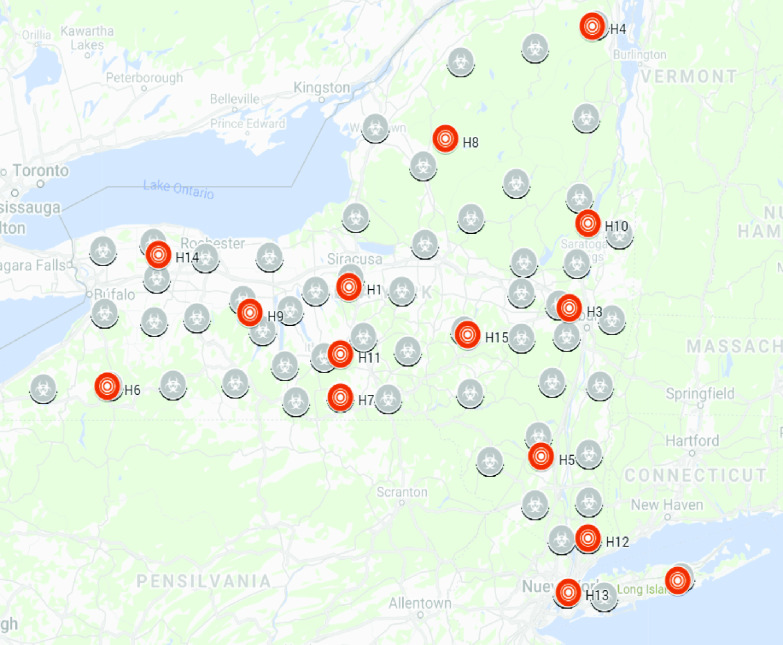
Hot spots $H_{k}$ for each cluster in New York state (orange circles).

The KDE (3) function is applied to the latitude (lat) and longitude (long) features of the data of each $c_{k}$ cluster. Fig. 6 shows the surfaces obtained with the KDE function for clusters $k = 1$ to $k = 4$. The obtained hot spots $H_{k}$ are shown in Fig. 7 (orange circles).

These $H_{k}$ hot spots were stored for further analysis in the following algorithm stages.

### C. Phase 3: Survival Rate Calculation for Each $k$ Cluster

In this phase, the survival rate of COVID-19 was determined for each cluster generated in Phase 1. This survival rate is important to determine the areas where medical attention and health resources are necessary to prevent and reduce deaths.

To calculate the survival rate, the information about the number of deaths dt of each cluster was used. The Cox proportional hazards model (CPHM), which gives the time $t$ that elapses before a death occurs, was applied. The survival function is denoted by $S(t)=Pr(T > t)$, where $S(t)$ is the probability that the random variable $T$ is larger than a specified time $t$, i.e., it represents the probability of an individual to survive up to time $t$ [23], [24].

The individual probability of hazard function $\lambda (t)$ is defined by

\begin{equation*} \lambda (t)=\displaystyle \lim _{ \delta \to 0}{\frac {{\mathrm{Pr}}(t \leq T+t+\delta |T \geq t)}{\delta }} \tag{4}\end{equation*}

This hazard function is a measure of risk at time $t$. A larger value means a greater risk of failure. It is composed of two functions: a baseline hazard function $\lambda _{0} (t)$ and a risk function $h(dt)$ denoting the effects of an individual’s covariates. The hazard function is assumed to have the form

\begin{equation*} \lambda (t|dt)=\lambda _{0}(t) e^{h(dt)} \tag{5}\end{equation*}

Applying (4) and (5) to each of the $k$ clusters, the survival rate and its priority is determined.

**FIGURE 8.**
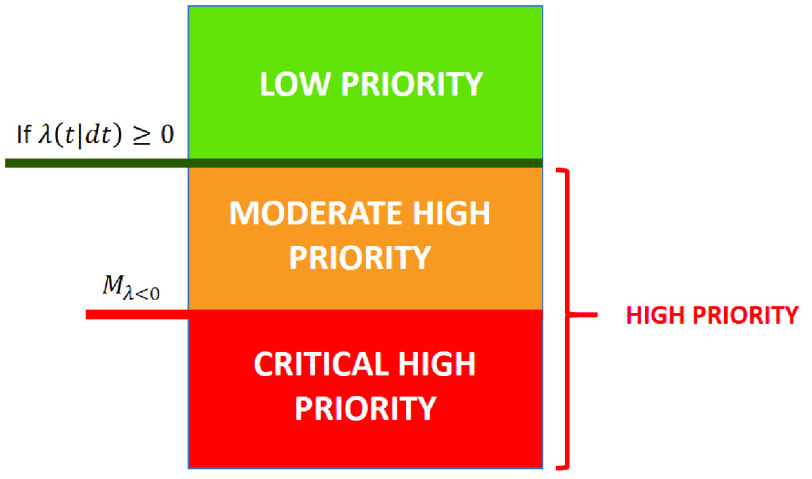
Hot spot priorities when applying the CPHM.

If $\lambda (t|dt) < 0$, it is classified as a high priority area with a high mortality rate, which requires urgent medical attention and more health resources. The mean of this value is $M_{\lambda < 0}$, which is used to divide it into two subgroups. If $\lambda (t|dt) < M_{\lambda < 0}$, the area is identified as critical high priority, and label Phc is assigned. If $\lambda (t|dt) \geq M_{\lambda < 0} < 0$, the area is considered moderate high priority, and labelled Phm. If $\lambda (t|dt)\geq 0$, the area is considered low priority, and label Pl is assigned, which means that health care is not as urgent as in the other areas but is still necessary to prevent the spread of the virus. The priority groups are shown in Fig. 8.

Table 4 shows the results of applying CPHM with the $\lambda (t|dt)$ values and the priority for each of the subgroups. Three subgroups were obtained. The first had two hot spots with priority Pl. For the other two subgroups, the mean of the values with high priority was calculated as $M_{\lambda < 0}= - 0.4037$. With this value, a subgroup with 10 hot spots with Phm priority and another subgroup with 3 hot spots with Phc priority were obtained.

**TABLE 4:** CPHM Results for Each $k$ Cluster and its Priority

$k$	1	2	3	4	5
$\lambda (t|dt)$	−0.482	−0.686	−0.26	−1.827	−0.146
Priority	Phc	Phc	Phm	Phc	Phm
k	6	7	8	9	10
$\lambda (t|dt)$	−0.146	−0.274	0.00	−0.359	−0.237
Priority	Phm	Phm	Pl	Phm	Phm
k	11	12	13	14	15
$\lambda (t|dt)$	0.00	−0.007	−0.021	−0.253	−0.402
Priority	Pl	Phm	Phm	Phm	Phm

These results were used to generate routes for health care according to priority.

### D. Phase 4: Health Care Routes Generation

This phase aims to identify the closest hospitals to each of the $H_{k}$ hot spots grouped according to their priority. In this way, optimal health care routes could subsequently be generated by applying ACO and TSP techniques.

The information about the hospitals and medical centers was determined by a vector of characteristics with the spatial location of each of the $m$ hospitals: hs$_{m}=\{$lat,long$\}$. To determine the closest hospital to the hot spots $H_{k}$ by their priority—Pl for low, Phm for moderate high, and Phc for critical high—the Euclidian distance between $H_{k}$ and hs_*m*_ is used, as defined by [25]

\begin{equation*} d(H_{k},{hs}_{m})=\sqrt {(x_{k}-x_{m})^{2}+(y_{k}-y_{m})^{2}} \tag{6}\end{equation*}

Then, the shortest distance $d(H_{k}$,hs_*m*_) is selected for each subgroup, and the corresponding hospital is assigned to the subgroup according to its priority (Table 5).

**TABLE 5:** Closest Medical Centers According to Group Priority

Priority	Latitude	Longitude	Hospital Name
Pl	41.057717	−74.768386	Newton Medical Center-New Jersey
Phm	40.764181	−73.956225	Memorial Sloan-Kettering Cancer Center
Phc	40.78	−72.97729	Long Island Community Hospital

Both ACO and TSP algorithms were applied to these three data subgroups to identify the most optimal routes for each one (i.e., the shortest route, minimum number of iterations, and less data).

#### 1) Application of ACO

The ACO algorithm performs several interactions that build solutions through the use of heuristic information. These algorithms use ants that collect experiences (pheromones) for future ant populations. Pheromones represent the trail each ant follows to find a solution (path). The ACO algorithm applies the pheromone update rule procedure, where an ant is a simple computational agent that interactively builds a solution to the problem. For each interaction performed by the algorithm, each ant moves from one status $r$ to another status $s$, obtaining a more complete intermediate solution [26], [27]. The $k^{th}$ ant from state r to state $s$ is selected among the unvisited states memorized in $J_{r}^{k}$:

\begin{equation*} s=arg_{\mu \in j_{r}^{k}}{{max}[\tau _{i}(r,\mu)^{\infty }\eta (r,\mu)^\beta]\quad if (q\leq q_{0})} \tag{7} \end{equation*}

The trail level represents a posteriori indication of the desirability of that move. Trails are usually updated when all ants have completed their solution. The trail is increased or decreased if that movement was part of the good or bad solution, respectively. The probability of the $k^{th}$ ant to move from state $r$ to state $s$ is

\begin{align*} p_{k} (r,s)= \begin{cases} \frac {\tau (r,s)^\infty \eta (r,s)^{\beta}} {\sum _{\mu \in J_{k}^{r}}{\tau (r,s)^\infty \eta (r,s)^\beta }}& if ~(s \in J_{r}^{k})\\ 0 & {\mathrm {otherwise}} \end{cases} \tag{8}\end{align*}

where $p_{k}(r, s)$ is the transition probability, $\tau(r, \mu)$ is the pheromone concentration between the state $r$ and the state $\mu$ of the $i^{t h}$ population, $\eta(r, \mu)$ is the length of the trail from the state $r$ and the state $\mu, J_{r}^{k}$ is the set of unvisited states of the $k^{t h}$ ant in the $i^{t h}$ population, $\alpha$ and $\beta$ are the control parameters, and $q$ is a uniform probability [0,1].

The solution will improve each time the trace of the pheromones is updated using

\begin{equation*} \tau (r,\mu)=(1- \rho)\tau (r,s)+\sum _{k=1}^{m} \Delta \tau _{k} (r,s) \tag{9} \end{equation*}

where $\rho (0 < \rho < 1)$ is the pheromone trail evaporation rate. In (9), $\Delta \tau _{k} (r,s)$ is the amount of pheromone trail added to the edge $(r,s)$ by ant $i$ between time $t$ and $t+\Delta t$, calculated by

\begin{align*} \Delta \tau _{i} (r,s)= \begin{cases}\dfrac {Q}{L_{i}}&(r,s)\in \pi _{i}\\ 0&{\mathrm {otherwise}}\end{cases} \tag{10}\end{align*}

where $Q$ is a constant parameter, and $L_{i}$ is the distance of the sequence $\pi _{t}$ toured by the ant in $\Delta t$.

#### 2) Application of the TSP Algorithm

The TSP algorithm, which determines the shortest route between a list of cities and distances, was also applied. This algorithm applies combinatorial optimization [28]. The TSP can be represented by a complete directed graph $G=(N,A)$, where $N$ is a set of $n$ nodes (vertices), also called cities; $A$ is a set of arcs; and $D=d_{ij}$ is the cost (distance) matrix associated with each arc $(i,j) \in A$, where $D$ can be either symmetric or asymmetric. The main objective of TSP is to find the shortest closed tour visiting each of the $n=|N|$ nodes of $G$. The TSP is defined by

\begin{align*} X_{ij}=\begin{cases}{1}&~\text{if the arc}(i,j)~\text{is in the tour}\\ 0&{\mathrm {otherwise}}\end{cases} \tag{11} \end{align*}

The TSP can be formulated by following the well-known integer program formulation, where $z$ is the objective function that represents the total cost to be minimized:

\begin{equation*} z={\mathrm{min}} \sum _{i} \sum _{j}^{d_{ij} x_{ij}} \tag{12} \end{equation*}

with the following constraints:

\begin{align*} \sum _{i-1}^{n} x_{ij}=&1,\quad j=1,2,3,\ldots,n \tag{13} \\ \sum _{j=1}^{n} x_{ij}=&1,\quad i=1,2,3,\ldots,n \tag{14}\\ x_{ij}\in&{0,1},\quad i,j=1,2,3,\ldots,n \tag{15} \\ \sum _{i,j \in S}^{n} x_{ij}\leq&|S|-1,\quad 2 \leq |S|\leq N-2 \tag{16} \end{align*}

The first constraint (13) ensures that each position $j$ is occupied by only one city, and the second constraint (14) guarantees that each city (node) $i$ is assigned to exactly one position. The third constraint (15) represents the integrality constraints of zero-one variables $x_{ij}~(x_{ij} \geq 0)$. The last constraint (16) ensures that each city (node) in the final route will be visited one time and that no sub-routes will be formed.

For the generation of the routes, the TSP algorithm is applied to the three subgroups of data according to their priority: Pl, Phm, and Phc [29].

## V. Experiment Results

This section presents the results obtained with the IIDA algorithm, that is, the shortest route to the closest medical center obtained for each subgroup. The experiments were performed using the Matlab R2019 version 5 software on a Pentium CPU i7 (8th generation) with 32.0 GB of RAM and Windows 10 64-bit operating system. For training, 3064 records were used, and 1276 were used for testing. The data for the tests were grouped into Test 1 (406 records from May 10 to May 16, 2020), Test 2 (406 records from May 17 to May 23, 2020), and Test 3 (464 records from May 24 to May 31, 2020).

To ensure a fair comparison, the simulations of the ACO and TSP route optimization algorithms have been executed with the same input values and following the same procedure. The input information is the three data subgroups according to their priority (Low Priority Pl, High Moderate Priority Phm, and Critical Priority Phc), and the location of the closest hospital for each of the subgroups, hs_*m*_. Subsequently, the executions have been carried out individually for each subgroup, and the optimal routes have been obtained.

Several techniques have been used in the different phases of the proposed algorithm. The configuration parameters of these methods have been determined based on an analysis of different configurations to ensure the robustness of the proposed values. For example, the elbow method has been applied to select the optimal number of clusters in which the infection sites are spatially classified. A range of values from $k = 1$ to $k = 55$ has been tested, and for each of the three data sets used, the most appropriate value has been obtained.

Additionally, several simulations were performed to determine the optimal configuration of the parameters of the ACO and TSP algorithms. For each one of them, a range of values was applied to the initial parameters, and the optimal values were identified. In the case of the ACO algorithm, values were tested for the number of ants between 10 and 200, pheromone factor $\alpha $ between 0.1 and 1, heuristic factor $\beta $ between 2.00 and 4.00, volatility coefficient $\rho $ from 0.1 to 1.00, and pheromone amount $\theta $ from 1 to 100. Besides, initial concentration values from 0.1 to 1 and maximum interaction $T$ between 1 and 200 were evaluated. The best values have been selected and are shown in Table 6. Likewise, the selected TSP algorithm configuration parameters are shown in Table 7. The maximum number of iterations MaxIter has been evaluated between 1 and 2000, and the initial popSize population from 1 to 500. For the execution of the simulations of the ACO and TSP algorithms, the initial configurations for each algorithm have been applied, as shown in Table 6 for ACO and Table 7 for TSP.

**TABLE 6:** ACO Algorithm Initial Parameters Configuration

Details	Values
Ants ($k$)	50
Pheromone factor $\alpha $	1
Heuristic factor$\beta $	4
Volatility coefficient $\rho $	0.2
Pheromone amount $\varphi $	100
Initial concentration $\tau _{ij}(0)$	1
Maximum iteration $T$	100

**TABLE 7:** TSP Algorithm Initial Parameters Configuration

Parameter	Values
Maximum number of iteration (MaxIter)	1000
Population Size	100

**FIGURE 9.**
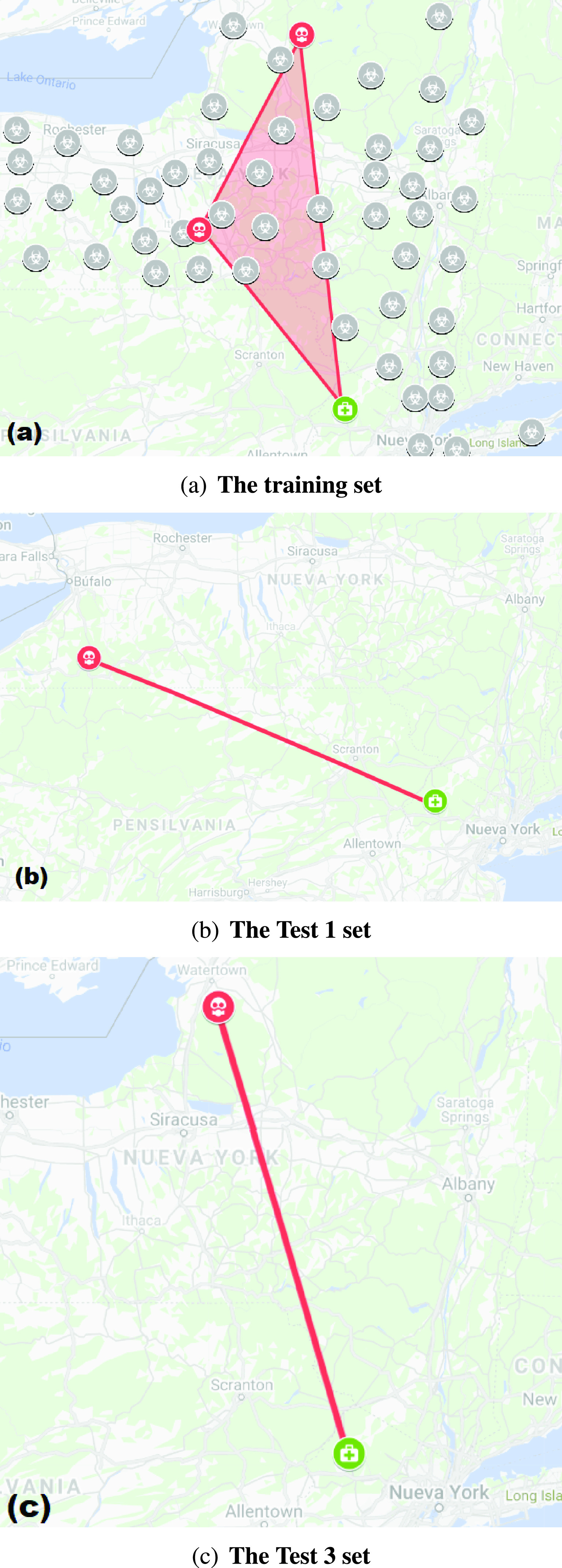
The route obtained with the ACO algorithm for low priority groups (Pl).

Fig. 9 shows the routes for low priority groups. The route optimization algorithm (ACO or TSP) that gave the best result in terms of time and route was selected in each case.

In Fig. 9a, two hot spots (orange circles) with low priority (Pl) (high survival rate) can be seen using the training set. The route is a triangle with a shaded area, which connects the closest hospital (green hospital icon) to the two hot spots. Fig. 9b shows the only hot spot of the Test 1 set that has low priority (orange circle) and the route to the closest hospital (green hospital icon). Fig. 9c shows the only hot spot that has low priority for the Test 3 dataset (orange circle) and the route to the hospital (green hospital icon). The Test 2 dataset did not have any hot spots with low priority Pl.

The simulation results are presented in Tables 8, 9, and 10: the column labeled BKS shows the length of the best-known solution obtained with the TSP and ACO algorithms. The column labeled Best shows the length of the best solution found for each algorithm. The column PDAv (%) is the percentage deviation of the average solution length over the best-known solution length, BKS (17).

\begin{equation*} {{\mathrm {PDAv}}} (\%)=\frac {{\mathrm {Average-{BKS}}}}{{\mathrm {BKS}}} \cdot 100 \tag{17} \end{equation*}

**TABLE 8:** ACO Algorithm Application Results

Data	k	# Points	BKS	Maximum	Minimum	Average	Best	S. dev.	PDav (%)	PDbest (%)	CPU Time
3064	15	18	3084	3101.72	3084	3092.83	3084	4,86	0.29	0.00	9.37
406	10	13	2299	2303.45	2299.12	2301.28	2299.12	1.13	0.1	0.01	5.99
406	9	11	1836	1840.69	1836.21	1838.45	1836.21	1.33	0.13	0.01	6.52
464	9	12	1815	1821.64	1815.09	1818.36	1815.09	1.95	0.19	0.00	7.25

**TABLE 9:** TSP Algorithm Application Results

Data	k	# Points	BKS	Maximum	Minimum	Average	Best	S. dev.	PDav (%)	PDbest (%)	CPU Time
3064	15	18	3084	3090.71	3084	3087.35	3084	1.72	0,11	0.00	9.6
406	10	13	2299	2302.45	2299.12	2300.78	2299.12	0.89	0.08	0.01	9.56
406	9	11	1836	1840.97	1836.1	1838.53	1836.1	1.56	0.14	0.01	5.16
464	9	12	1815	1820.67	1815.02	1817.84	1815.02	1.55	0.16	0.00	5.8

**TABLE 10:** IIDA Proposed Algorithm General Results

Data	Best (km)	Worst (km)	PDav(%)	PDbest (%)	CPU Time
3064	3084	3101.72	0.11	0.00	23.86
406	2299.12	2303.45	0.08	0.01	18.58
406	1836.1	1840.97	0.13	0.01	14.83
464	1815.02	1821.64	0.16	0.00	16.25

PDBest (%) calculates the percentage deviation of the length of the best solution for each algorithm, Best, over the best-known solution length, BKS (18).

\begin{equation*} {{\mathrm {PDBest}}} (\%)=\frac {{\mathrm {Best-{BKS}}}}{{\mathrm {BKS}}} \cdot 100 \tag{18} \end{equation*}

The average of the results obtained in 10 runs for each data set with the ACO and the TSP algorithms are presented in Table 8 and Table 9, respectively. The final results of the routes obtained with the IIAD algorithm are shown in Table 10.

## VI. Discussion

From the results shown in Table 10, it can be concluded that the phases of the algorithm are well designed when the objective is to determine optimal healthcare routes based on spatial hotspots of infected people. Both the ACO and TSP algorithms yield very good results (Tables 8 and 9). In fact, the PDAv values are between 0.10% and 0.29% for ACO and between 0.08% and 0.16% for TSP. The percentage deviation of the length for the best solution for both, ACO and TSP, is very close to 0.00%, which demonstrates the efficiency of these algorithms in this application. These values mean that the deviation from the best route solution is very small in any case. On the other hand, the PDBest(%) reaches also very good results, up to 0.01%.

In Tables 8 and 9, the number of points (that is, the way-points of the route) has a direct relationship with PDBest (%). The greater the number of spatial points to visit, the better the route solution obtained with both, ACO and TSP algorithms and not necessarily the larger one. In addition, standard deviation is very low (Table 8 and 9), which indicates that most of the results obtained tend to be grouped close to their mean, thus giving an optimal solution.

The length of the route is different for each data sets, even if they have the same number of infection records. For example, dataset Test 2 and dataset Test 3 have 406 infections records, and the routes are 2299.12 km 1836.10 km respectively, which agrees with the number of way-points.

Regarding the efficiency of the IIDA algorithm, the maximum PDAv is 0.16% and the maximum PDBest (%) is 0.01%. This shows that the generated routes are very efficient, although the computational time is high, between 16.25 seconds and 23.86 seconds.

Some similar proposals found in the literature are the research of Nelli *et al.* (2020) [12] and Kang *et al.* (2020) [15]. In Nelli (2020), a model is presented to predict the probability of Malaria infection in eight rural clinics, based on the road travel distances from the surrounding villages (Burkina Faso). The results obtained for infection prediction are good, reaching up to 100% accuracy. Kang’s work explores the Covid-19 spatial epidemic dynamics in mainland China, applying Moran’s I spatial statistic. Very good results are obtained with regard to identifying infection areas and their spatial association.

The proposed algorithm uses information that allows routes to be prioritized. It calculates the mortality rate, which makes it possible to identify sectors with the greatest need for health resources. This can be an advantage when monitoring virus spread compared to the other research mentioned above.

## VII. Conclusion and Future Research

In this work, the Intelligent Infectious Disease Algorithm IIDA has been developed to identify the main infection sources (hot spots) of COVID-19 by applying k-means clustering (with the elbow method) and the statistical function KDE. The algorithm estimates the survival rate of each of the hot spots by applying the proportional hazards model. With this survival rate, a priority is assigned for the generation of routes to the closest medical center. These heath care routes are generated by applying the evolutionary ACO and TSP algorithms. It has been applied to New York state.

The proposed IIDA algorithm can improve the health care response time to a pandemic like Covid-19 by determining areas with higher infection rates and mortality. The IIDA performs a spatial distribution of the infection sources from the analyzed information, which allows us to determine the optimal routes for medical care within a reasonable processing time.

The number of clusters determined by the elbow method is relevant since it represents the number of hotspots and thus the way points of the healthcare routes. For this reason, the number of clusters can be fine-tuned to improve spatial coverage in small regions with isolated infections.

Although the routes obtained using the ACO algorithm are good, the processing time of the entire algorithm is high for its execution in real time. A possible extension of the work would be to optimize the code and its implementation in distributed systems to reduce computational time.

As future research, it is proposed to incorporate temporary information on virus spread. Spread models could also be completed with other types of information related to space, such as the social and cultural environment of the region. Regarding other methodologies, beyond using one technique or another, the Moran’s I method could be applied to determine the spatial autocorrelation between different regions with infected people.

During this study, the importance of including the infection rate and the incubation time to determine time periods for the analysis of disease spread became evident.

## References

[1] Coronavirus Disease (COVID-19). Accessed: Oct. 29, 2020. [Online]. Available: https://www.who.int/emergencies/diseases/novel-coronavirus-2019

[2] A. I. Albarrak, R. Mohammed, A. Al Elayan, F. Al Fawaz, M. Al Masry, M. Al Shammari, and S. B. Miaygil, “Middle east respiratory syndrome (MERS): Comparing the knowledge, attitude and practices of different health care workers,” J. Infection Public Health, to be published, doi: 10.1016/j.jiph.2019.06.029. PMC7102554 31431424

[3] D. P. Kavadi, R. Patan, M. Ramachandran, and A. H. Gandomi, “Partial derivative nonlinear global pandemic machine learning prediction of COVID 19,” Chaos, Solitons Fractals, vol. 139, Oct. 2020, Art. no. 110056. 10.1016/j.chaos.2020.110056 PMC7315984 32834609

[4] C. Zhang, Z. Du, Q. Cai, L. Yu, Z. Li, and Y. Bai, “Assignment optimization of pandemic influenza antiviral drugs in urban pharmacies,” J. Ambient Intell. Humanized Comput., vol. 10, no. 8, pp. 3067–3074, Aug. 2019.

[5] A. M. Kramer, J. T. Pulliam, L. W. Alexander, A. W. Park, P. Rohani, and J. M. Drake, “Spatial spread of the West Africa Ebola epidemic,” Roy. Soc. Open Sci., vol. 3, no. 8, Aug. 2016, Art. no. 160294. 10.1098/rsos.160294 PMC5108957 27853607

[6] A. F. Y. Poon, R. Gustafson, P. Daly, L. Zerr, S. E. Demlow, J. Wong, C. K. Woods, R. S. Hogg, M. Krajden, D. Moore, P. Kendall, J. S. G. Montaner, and P. R. Harrigan, “Near real-time monitoring of HIV transmission hotspots from routine HIV genotyping: An implementation case study,” Lancet HIV, vol. 3, no. 5, pp. e231–e238, 5 2016. 27126490 10.1016/S2352-3018(16)00046-1 PMC4853759

[7] S. Gryseels, S. J. E. Baird, B. Borremans, R. Makundi, H. Leirs, and J. G. de Bellocq, “When viruses don’t go viral: The importance of host phylogeographic structure in the spatial spread of arenaviruses,” PLOS Pathogens, vol. 13, no. 1, Jan. 2017, Art. no. e1006073. 10.1371/journal.ppat.1006073 PMC5226678 28076397

[8] A. Wesolowski, E. Z. Erbach-Schoenberg, A. J. Tatem, C. Lourenço, C. Viboud, V. Charu, N. Eagle, K. Engø-Monsen, T. Qureshi, C. O. Buckee, and C. J. E. Metcalf, “Multinational patterns of seasonal asymmetry in human movement influence infectious disease dynamics,” Nature Commun., vol. 8, no. 1, pp. 1–9, Dec. 2017. 29234011 10.1038/s41467-017-02064-4 PMC5727034

[9] G. Guzzetta, C. A. Marques-Toledo, R. Rosà, M. Teixeira, and S. Merler, “Quantifying the spatial spread of dengue in a non-endemic Brazilian metropolis via transmission chain reconstruction,” Nature Commun., vol. 9, no. 1, pp. 1–8, Dec. 2018. 30026544 10.1038/s41467-018-05230-4 PMC6053439

[10] Z. Li, J. Fu, G. Lin, and D. Jiang, “Spatiotemporal variation and hotspot detection of the avian influenza A(H7N9) virus in China, 2013–2017,” Int. J. Environ. Res. Public Health, vol. 16, no. 4, p. 648, Feb. 2019. 10.3390/ijerph16040648 PMC6406651 30813229

[11] M. Kate Grabowski, J. Lessler, J. Bazaale, D. Nabukalu, J. Nankinga, B. Nantume, J. Ssekasanvu, S. J. Reynolds, R. Ssekubugu, F. Nalugoda, G. Kigozi, J. Kagaayi, J. S. Santelli, C. Kennedy, M. J. Wawer, D. Serwadda, L. W. Chang, and R. H. Gray, “Migration, hotspots, and dispersal of HIV infection in Rakai, Uganda,” Nature Commun., vol. 11, no. 1, pp. 1–12, Dec. 2020. 32080169 10.1038/s41467-020-14636-y PMC7033206

[12] L. Nelli, M. Guelbeogo, H. M. Ferguson, D. Ouattara, A. Tiono, S. N’Fale, and J. Matthiopoulos, “Distance sampling for epidemiology: An interactive tool for estimating under-reporting of cases from clinic data,” Int. J. Health Geographics, vol. 19, no. 1, pp. 1–14, Apr. 2020. 10.1186/s12942-020-00209-1 PMC7171748 32312266

[13] E. L. Ray and N. G. Reich, “Prediction of infectious disease epidemics via weighted density ensembles,” PLOS Comput. Biol., vol. 14, no. 2, Feb. 2018, Art. no. e1005910. 10.1371/journal.pcbi.1005910 PMC5834190 29462167

[14] G. M. Nandana, S. Mala, and A. Rawat, “Hotspot detection of dengue fever outbreaks using DBSCAN algorithm,” in Proc. 9th Int. Conf. Cloud Comput., Data Sci. Eng. (Confluence), Jan. 2019, pp. 158–161, doi: 10.1109/CONFLUENCE.2019.8776916.

[15] D. Kang, H. Choi, J.-H. Kim, and J. Choi, “Spatial epidemic dynamics of the COVID-19 outbreak in China,” Int. J. Infectious Diseases, vol. 94, pp. 96–102, 5 2020. 32251789 10.1016/j.ijid.2020.03.076 PMC7194591

[16] A. d’Onofrio, M. Banerjee, and P. Manfredi, “Spatial behavioural responses to the spread of an infectious disease can suppress turing and Turing–Hopf patterning of the disease,” Phys. A, Stat. Mech. Appl., vol. 545, 5 2020, Art. no. 123773.

[17] I. Sung and T. Lee, “Optimal allocation of emergency medical resources in a mass casualty incident: Patient prioritization by column generation,” Eur. J. Oper. Res., vol. 252, no. 2, pp. 623–634, Jul. 2016.

[18] I. Sumaiya Thaseen and C. Aswani Kumar, “Intrusion detection model using fusion of chi-square feature selection and multi class SVM,” J. King Saud Univ.-Comput. Inf. Sci., vol. 29, no. 4, pp. 462–472, Oct. 2017.

[19] M. Sharma, “Improved autistic spectrum disorder estimation using Cfs subset with greedy stepwise feature selection technique,” Int. J. Inf. Tecnol., pp. 1–11, Jul. 2019, doi: 10.1007/s41870-019-00335-5.

[20] J. Rojas-Thomas, M. Santos, M. Mora, and N. Duro, “Performance analysis of clustering internal validation indexes with asymmetric clusters,” IEEE Latin Amer. Trans., vol. 17, no. 05, pp. 807–814, 5 2019.

[21] R. Fathi, A. Mohammed, and H. Hefny, “Spatial clustering and analysis on hepatitis C virus infections in Egypt,” Int. J. Data Mining Knowl. Manage. Process, vol. 8, nos. 4–5, pp. 01–13, Sep. 2018.

[22] T. M. Davies, C. R. Flynn, and M. L. Hazelton, “On the utility of asymptotic bandwidth selectors for spatially adaptive kernel density estimation,” Statist. Probab. Lett., vol. 138, pp. 75–81, Jul. 2018.

[23] B.-H. Min, C. H. Tae, S. M. Ahn, S. Y. Kang, S.-Y. Woo, S. Kim, and K.-M. Kim, “Epstein-barr virus infection serves as an independent predictor of survival in patients with lymphoepithelioma-like gastric carcinoma,” Gastric Cancer, vol. 19, no. 3, pp. 852–859, Jul. 2016. 26265391 10.1007/s10120-015-0524-x

[24] F. Emmert-Streib and M. Dehmer, “Introduction to survival analysis in practice,” Mach. Learn. Knowl. Extraction, vol. 1, no. 3, pp. 1013–1038, Sep. 2019.

[25] R. Naranjo, M. Santos, and L. Garmendia, “A convolution-based distance measure for fuzzy singletons and its application in a pattern recognition problem,” Integr. Comput.-Aided Eng., to be published.

[26] Y. Zhang, H. Zhao, Y. Cao, Q. Liu, Z. Shen, J. Wang, and M. Hu, “A hybrid ant colony and cuckoo search algorithm for route optimization of heating engineering,” Energies, vol. 11, no. 10, p. 2675, Oct. 2018.

[27] W. Deng, J. Xu, and H. Zhao, “An improved ant colony optimization algorithm based on hybrid strategies for scheduling problem,” IEEE Access, vol. 7, pp. 20281–20292, Feb. 2019.

[28] M. Cárdenas-Montes, “Creating hard-to-solve instances of travelling salesman problem,” Appl. Soft Comput., vol. 71, pp. 268–276, Oct. 2018.

[29] E. Osaba, R. Carballedo, F. Diaz, E. Onieva, A. D. Masegosa, and A. Perallos, “Good practice proposal for the implementation, presentation, and comparison of metaheuristics for solving routing problems,” Neurocomputing, vol. 271, pp. 2–8, Jan. 2018.

